# “We Want to Talk about Death, Dying and Grief and to Learn about End-of-Life Care”—Lessons Learned from a Multi-Center Mixed-Methods Study on Last Aid Courses for Kids and Teens

**DOI:** 10.3390/children11020224

**Published:** 2024-02-09

**Authors:** Georg Bollig, Kirsti Gräf, Harry Gruna, Daniel Drexler, Raymund Pothmann

**Affiliations:** 1 Department of Anesthesiology, Intensive Care, Palliative Medicine and Pain Therapy, Helios Klinikum, 24837 Schleswig, Germany; 2 Department of Palliative Medicine, Faculty of Medicine and University Hospital, University of Cologne, 50924 Cologne, Germany; 3 Last Aid Research Group International (LARGI), 24837 Schleswig, Germany; 4 Letzte Hilfe Deutschland gGmbH, 24837 Schleswig, Germanyharrygruna@t-online.de (H.G.); danieldrexler@posteo.de (D.D.); raymund.pothmann@kinderpact-hamburg.de (R.P.); 5 Pfeiffersche Stiftungen, 39114 Magdeburg, Germany; 6 Department of Palliative Medicine, RoMed Klinikum, 83022 Rosenheim, Germany; 7 Kinder PACT, 22297 Hamburg, Germany

**Keywords:** Last Aid Course kids/teens, awareness, death, dying, death literacy, palliative care, public palliative care education, children, teenager, mixed-methods

## Abstract

Background: Last Aid Courses (LAC) for adults have been established in 21 countries in Europe, Australia and America to improve the public discussion about death and dying and to empower people to participate in end-of-life care provision. In 2018, the first Last Aid Courses for kids and teens (LAC-KT) were introduced. The aim of the study was to explore the views and experiences of the course participants and Last Aid Course instructors on the LAC-KT. Methods: A mixed-methods approach was used. The views of the LAC-KT participants, aged 7 to 17 years, on the LAC-KT were collected using a questionnaire. In addition, the experiences of the Last Aid Course instructors were explored in focus group interviews. Results: The results show that 84% of the participants had experiences with death and dying and 91% found the LAC-KT helpful for everyone. The majority of the participants appreciate the opportunity to talk and learn about death, dying, grief and palliative care. Conclusions: The LAC-KT is feasible, very well accepted and a welcome opportunity for exchanging and obtaining information about dying, grief and palliative care. The findings of the study indicate that the LAC-KT should be offered to all interested children and teenagers and included in the school curriculum.

## 1. Introduction

The awareness of palliative care and the knowledge about end-of-life care is low among the public, and death-literacy is missing in general [1,2,3,4,5,6,7]. Due to the lack of awareness of palliative care, different experts have recommended education of the public [1,2,3,4,5,6,7,8]. The public needs knowledge about palliative care because palliative care is everyone’s business according to Kellehear [9], and the fact is that the need for palliative care in the community is rising [10]. The Lancet Commission on the Value of Death and other experts have recommended to improve death literacy, knowledge and skills in end-of-life care for everyone [1,2,3,4,5,6,7,8,11,12,13,14]. According to the World Health Organization’s Ottawa Charter for Health Promotion, the collaboration between health care services and communities should be an essential part of public health care, including end-of-life care [8,14]. Last Aid Courses have been established in many countries as a response to the growing need for palliative care everywhere with the aim to enhance the public discussion on death, dying and grief and to provide basic knowledge and skills in palliative care for the public [8,12,13]. Last Aid Courses may serve as the knowledge base for the public to enable and empower them to contribute to compassionate communities [8,9]. Along with the successful introduction of Last Aid Courses (LAC) for adults in Germany, Norway and Denmark in 2014–2015, the idea to adapt Last Aid Courses for children and teenagers was suggested by GB, some of the experienced LAC instructors and some palliative care experts. The discussion led to establishing a working group and the introduction of a special Last Aid Course for Kids and Teens (LAC-KT) aged 8 to 16 years in 2018 [8,13]. The main findings from a German pilot-study with 120 participants were that the course was feasible, very well accepted by children and teenagers and that many participants appreciated the possibility to talk openly about death, dying and to explore practical Last Aid measures that everybody can apply [13].

The aim of the present study was to research the experiences and views of the participating children and teenagers, between the age of 7–17 years, with the LAC-KT and the topics death, dying and grief among the Last Aid Course participants. In addition, the views of certified Last Aid Course instructors on the course, its practical aspects and implementation were also studied. 

The results from the current study may inform the public, health-care personnel, researchers, politicians and decision-makers. Both the LAC and LAC-KT may contribute to reduce the death taboo in society. Furthermore, the results can inform people who are working with the implementation of caring communities and the improvement of palliative care in the communities.

## 2. Materials and Methods

### 2.1. The Concept and Curriculum of the Last Aid Course for Kids and Teens (LAC-KT)

The international Last Aid Course for adults (LAC), introduced in 2015, is grounded in a standardized curriculum as the result of a consensus of a multi-professional working group consisting of international palliative care experts [8,12]. Based on the LAC for adults, a working group of experts from the fields of palliative care and education adapted the LAC to the needs of children and teenagers between 8 and 16 years of age. Both the LAC and the LAC-KT consist of four modules, each delivered over 45 min. The main themes of the four modules o are: 1. Dying as a normal part of life; 2. Planning ahead; 3. Relieving suffering; and 4. Final goodbyes. The LAC-KT is more interactive in general and uses activities and videos to suit the needs and wishes of children and teenagers. Children and teenagers who participated in the first pilot-courses gave both written and oral feedback on the first pilot-concept and curriculum. Based on their feedback and preferences, the course contents and the curriculum were adapted.

The LAC-KT includes the following videos in the German language, which can be viewed free online (links provided in the references):

1. The old badger; about dying and grief suited for children and teenagers [15].
2. Bill’s story; about compassionate communities and what everyone in the community may contribute [16].
3. Knietzsche und der Tod (Knietzsche and death); about philosophical views on death and dying from the perspective of a child philosopher [17].
4. Sowieso; a music video from Mark Forster as farewell at the end of the course [18].

Usually, a short break follows every module of the course. The course lasts between 3.5 and 4 h, including the breaks, if delivered over one day. The LAC-KT was delivered by two certified instructors. One of the instructors must have practical experience in the field of palliative care. The working group who established the curriculum and presentation for the LAC-KT defined the target group for the course as children and teenagers aged between 8 and 16 years. The participants of the pilot courses recommended not excluding younger or older children from participation in the LAC-KT. However, as school classes and other groups also include older and younger members, the age was adjusted to between 7 and 17 for inclusion in the current study. Older participants were allowed to participate in some of the courses, but they were excluded as informants for the present study. Table 1 shows the contents of the Last Aid Course for children and teenagers, usually referred to as the Last Aid Course for Kids and Teens (LAC-KT).

### 2.2. Setting and Participants

All participating Last Aid Course instructors had certification as an instructor for Last Aid Courses for kids and teens. All certified instructors had successfully completed a one day instructor course provided by Letzte Hilfe Deutschland gGmbH, Schleswig, Germany [20]. The LAC-KT instructors were responsible for both informing people about the course and the accompanying research project and for conducting the LAC-KT. They approached people in schools, churches and other organizations to recruit participants for the LAC-KT and the study. The LAC-KTs that were included in the study were held in different parts of Germany, hosted by different organizations, such as ambulatory hospice services, palliative care services, schools and churches. The inclusion criteria for informants in this study were children and teenagers aged between 7 and 17 years old who had participated in an LACV-KT. Exclusion criteria for informants were being aged under 7 or over 17 years and not having attended the whole LAC-KT course. All LAC-KTs were held in a classroom-setting as the course is not provided as an online course. The expert working group that established the course and the instructors agreed not to conduct an online course because of the lack of group activities and the lacking possibility to intervene if participants should need active support. Therefore, no courses were held in the lock-down periods during the COVID-19 pandemic.

### 2.3. Data Collection and Analysis

The study used a mixed-methods methodology [21] combining the collection of quantitative and qualitative data from a questionnaire for the LAC-KT participants and focus group discussions for the LAC-KT instructors. The questionnaire included both quantitative and qualitative parts. The questionnaire for the participants is available in Table S1 as part of the supplementary material. A mixed-methods approach was used in order to provide a bigger picture of the topic. Focus-group discussions were used to explore the instructors’ views in-depth. All participants were invited to provide their feedback using a paper questionnaire and were informed that filling out the questionnaire was voluntary. The questionnaire was distributed during the course. The quantitative data from the questionnaire were analyzed and described through quantitative description. The focus group interviews were led by a researcher with experience in qualitative research with the help of a second researcher. A semi-structured guide for the focus group interviews included the following questions to start the group discussion:

- What are your experiences with Last Aid Courses for Kids and Teens in general?
- What are your positive experiences with Last Aid Courses for Kids and Teens?
- What are your negative experiences with Last Aid Courses for Kids and Teens?
- Which methods did you use for practice and hands-on training in the Last Aid Courses for Kids and Teens?
- What are your views and experiences concerning communication with teachers and schools?

The interviews were held in a classroom setting with digital audio recording or online as a video meeting, digitally recorded and transcribed verbatim by a trained secretary. 

The data were collected on paper, scanned and stored electronically. All paper questionnaires were scanned and stored electronically on the researcher’s laptop after collection. Only the researchers on the project team had access to the data. To protect the privacy of the participants, no personal data other than age and gender were collected. The analysis of the collected qualitative data was based on qualitative content analysis and qualitative description [22,23,24,25]. Qualitative description served to provide rich and straightforward descriptions of the instructors’ and participants’ views collected from the focus-group discussions and the open questions from the questionnaire in everyday terms [22,23]. Qualitative description and qualitative content analysis were used to analyze the qualitative data using an inductive approach and a collection of data-derived themes [21,22,23,24,25]. GB and KG analyzed the qualitative data through repeated readings of the transcripts. Thereafter, they established preliminary codes from the data material. The preliminary codes were discussed by the researchers and revised in three discussion rounds. The last step of the qualitative data analysis was questioning the findings using meta-positions [25]. This was conducted by GB, KG and RP, who all have extensive experience in the analysis of qualitative data. The analysis process ended after all authors agreed both on the codes and themes found in the qualitative data material and the interpretation of the data.

### 2.4. Ethical Considerations and Ethics Approval

Informed consent was obtained from all participants. All participants received information about the purpose of the study prior to participation. All participating children, teenagers and parents were informed about the LAC-KT and the accompanying research through written information and/or information meetings before participation in the LAC-KT and the scientific evaluation. All had the possibility to ask questions prior to providing their consent to participate in the study. Informed consent was given by the parents (and the children and teenagers) after they had received the information. The LAC-KT participants in the study were asked to complete a questionnaire after participation in the course. The only task which study participants from the participant groups had to complete was to return a questionnaire about their views on the LAC-KT. Participation in the study was voluntary. The instructors were invited to participate in a focus discussion. The study described in this article is a part of larger research project that aims to evaluate the effects of Last Aid Courses. This project has been reported to the Regional Ethical Committee of Southern Denmark. The Regional Ethical Committee concluded that no formal application was required (The Regional Committees on Health Research Ethics for Southern Denmark; nr. 20182000-33). To protect the privacy of the participants, no personal data other than age and gender were collected. The informants could choose whether they wanted to participate in the study and wished to provide this information or not. In order to handle possible psychological reactions of course participants who might have had previous traumatic experiences with death and dying, the LAC-KT was always delivered by two people with experience in palliative care and grief. All instructors were capable of handling such possible reactions and to establish contact to other health care providers (e.g., grief experts or psychologists) that could assist if needed.

## 3. Results

The study started in March 2020. Due to the COVID-19 pandemic, classroom teaching was very restricted and sometimes impossible during some periods of the project. During the lockdown period, no LAC-KT courses were held. Between March 2020 and October 2023, 168 LAC-KTs were held in different regions and cities in Germany. The focus group interviews of the LAC-KT instructors were held in January and April 2023. 

### 3.1. Results from the Quantitative Data from the Questionnaire

**Figure 1:**
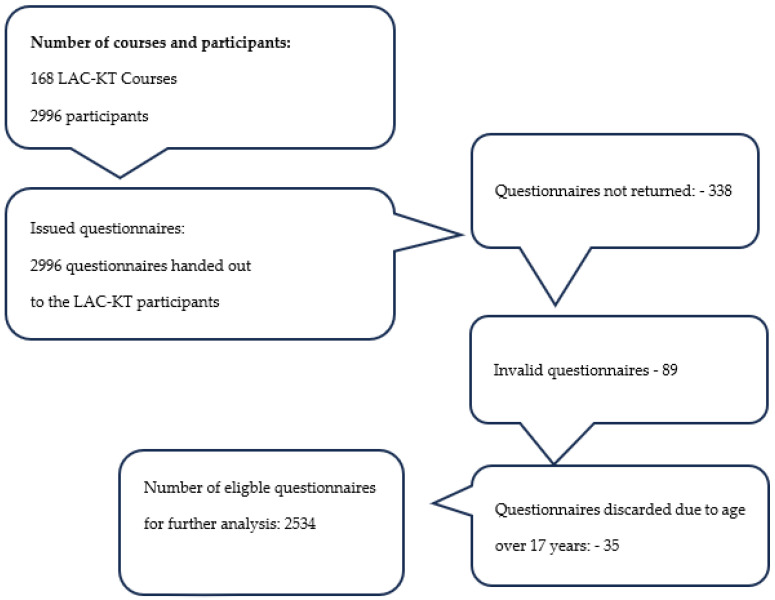
Overview of courses and included LAC-KT participants.

A total number of 2996 children and teenagers participated in the LAC-KT within the study period, and 2534 of them participated in the study by returning a questionnaire after participation in the LAC-KT (see Figure 1). 

The present study on Last Aid Courses for Kids and Teens is based on a total number of 2996 participants. Due to a complete pause during the COVID pandemic, most courses took place between mid-2022 and October 2023. The questionnaire was designed to provide basic information about the participants, including age, gender and the school class, as well as the participants´ feedback about the individual modules, the course format, their personal impression and suggestions for changes and improvement. The base for the study was from 2534 valid questionnaire responses. The overall response rate was 85%. 

**Figure 2:**
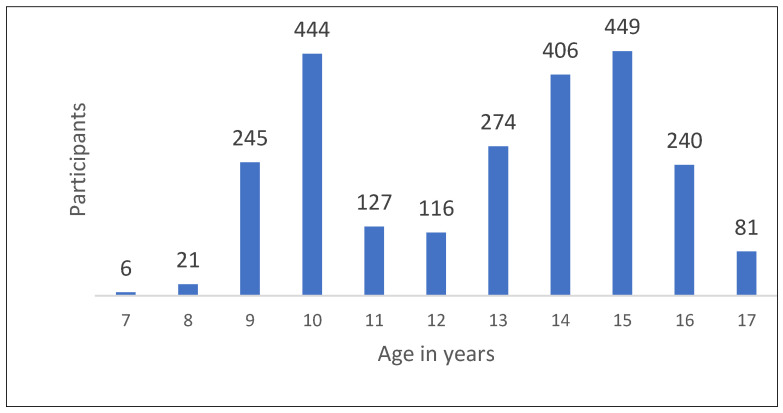
Age distribution of the LAC-KT participants (*n* = 2409).

**Figure 3:**
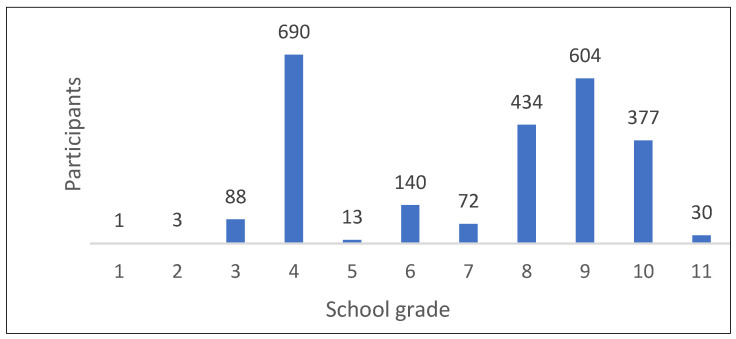
Classification of participants according to school grades (*n* = 2452).

The age distribution, as shown in Figure 2, included 2409 children and teenagers between 7 and 17 years of age. Meanwhile, 125 participants did not provide any information about their age. The average age of the participants was 12.8 years. Figure 3 includes data from the 2452 participants who provided information about their school grades. It shows the classification of the participants according to school grades.

**Figure 4:**
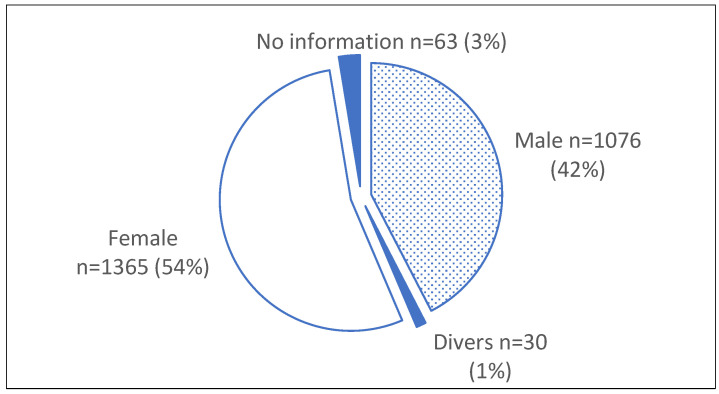
Gender distribution of LAC-KT participants (*n* = 2534).

Among all of the school grades, four grades clearly stood out. In the fourth grade, special topics are usually worked into the school curriculum before the end of primary school. In a number of German schools, the topic of death and dying is one of these special topics. In grades eight to ten, the school curriculum often includes so-called “Project Weeks”, in which a topic is dealt with in more depth. Participants of Confirmation classes in the Protestant church often come from the eighth and ninth grades. However, 82 participants did not provide any information about their school grades. Figure 4 illustrates the gender of the participants divided into three categories: female, male and non-binary. 

**Figure 5:**
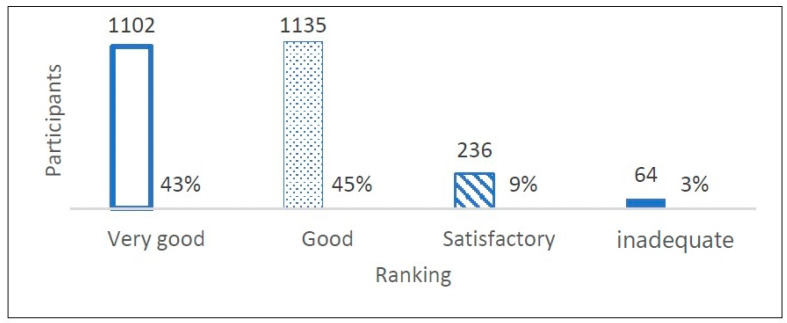
Participants’ ranking of the LAC-KT (*n* = 2534).

**Figure 6:**
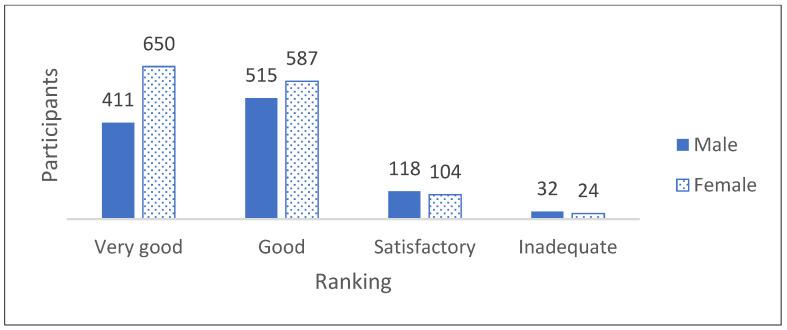
Ranking of the LAC-KT by gender (male and female *n* = 2441).

The data show that most of the participants were female (54%). However, 3% of the informants chose not to provide information on their gender. As part of the questionnaire for the course evaluation, the informants were asked to rate the course using the categories very good, good, satisfactory and inadequate. The results showed that the overall evaluation of the course format was rated as good or very good by 88% of the participants. Satisfactory to inadequate was stated by 12%, in relation to the total number *n* = 2534. This number illustrates the high degree of appreciation and acceptance of the LAC-KT by the participants. The ranking of the entire course by all of the informants is presented in Figure 5, whereas Figure 6 shows the ranking distributed by gender.

**Figure 7:**
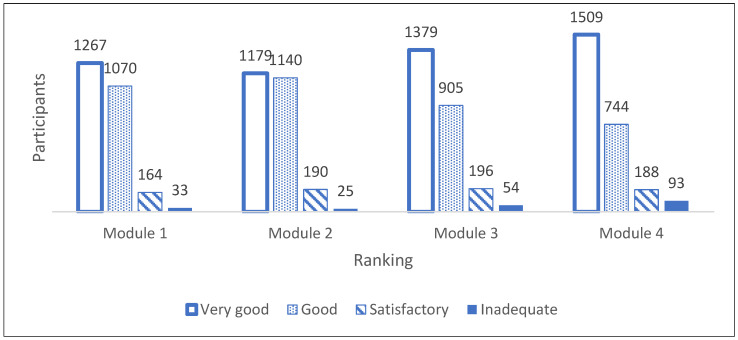
Ranking of the different modules by the participants (*n* = 2534).

When broken down into gender, it can be seen that female participants rated the course format better than male participants, while there were only minor differences in the four categories very good, good, satisfactory and inadequate. The group of non-binaries was not included in the figure because of the small number. The LAC-KT is divided into four modules: 1. Dying as a normal part of life; 2. Planning ahead; 3. Relieving suffering; and 4. Final goodbyes. Module 4 was rated best by the participants. The other three modules were rated close to each other in their evaluation. Module 2 deals with advance care planning and living wills, which, compared to Module 3 and 4, is rather theoretical, and was the least popular module in the data material (see Figure 7). Nevertheless, the rankings of all the modules are quite similar, with relatively small differences related to the size of the data sample.

As part of the questionnaire, the participants were asked about their personal impression of the course. Eight questions could be answered by ticking yes or no (see Table 2).

The LAC-KT is highly appreciated by the participants; for example, 91% found it helpful for everyone and 87% found the course contents easy to understand. Interestingly, 84% of the children reported experience with death and dying among family members or friends. 

### 3.2. Results from the Qualitative Data from the Questionnaire and the Focus Group Interviews

#### 3.2.1. Results from the Qualitative Data from the Questionnaire

All of the LAC-KT participants received the questionnaire and had the opportunity to write down what they particularly liked and to provide comments on every topic they wanted to. Not all of the informants used the possibility to write comments about the course or suggestions for improvement in their own language. The responses can be divided into the five categories that were the most frequently mentioned (see Table 3).

The short videos used in the LAC-KT were very well received by participants from all age groups. Most of the participants stated that the videos provided additional information or viewpoints and illustrated the topics they covered very well. The mention of the painting stones activity as an example of a helpful ritual shows that the creative handling of the topic of death and dying was of particular importance to the participants. The fact that movement and fun are important in the course format was evident in the nomination. Many of the participants liked playing with the swing cloth very much. Here, the topic of networking and cooperation of professionals and citizens was taught in a playful way. Mouth care practice is conducted in all courses as a practical exercise and was very much appreciated. The reason for this may be that the participants can do something practical for their relatives to relieve suffering and thirst at end-of-life. “Talking” had a broad spectrum in the mentions, from the openness to talking about the topic of death and dying, the given opportunity to talk about death and dying at all and that each participant could talk about death and dying as he or she wanted. Of course, it also included the fact that everyone was heard during the discussions. Another frequently mentioned point was that the children and young people were able to “talk” about death and dying in their respective familiar groups, in the sense of exchanging ideas about these usually uneasy topics. 

Many of the participants wrote comments about the course in the free space on the front- or back-side of the questionnaire. Most of the participants wrote that they do not have any open questions after participating in the LAC-KT. Many of the participants provided positive feedback and commented on what they liked best. Some examples are shown here:

*I liked module 3 best because I found it very interesting what oneself can do.*

(girl, 8 years)

*I liked very much that we have talked about it, because you do not talk about death very often.*

(girl, 13 years)

*I liked that we have talked extensively and open about life and death and that we also have talked about the feelings of dying people.*

(girl, 13 years)

*I liked everything.*

(boy, 9 years)

*Most of all I liked the videos and the practical activities.*

(boy, 14 years)

*I liked everything. The best theme was final goodbyes.*

(girl, 15 years)

*I liked best the network game and the mouth care.*

(girl, 9 years)

#### 3.2.2. Interview Findings from the Focus Group Interviews of LAC-KT Instructors

**Figure 8:**
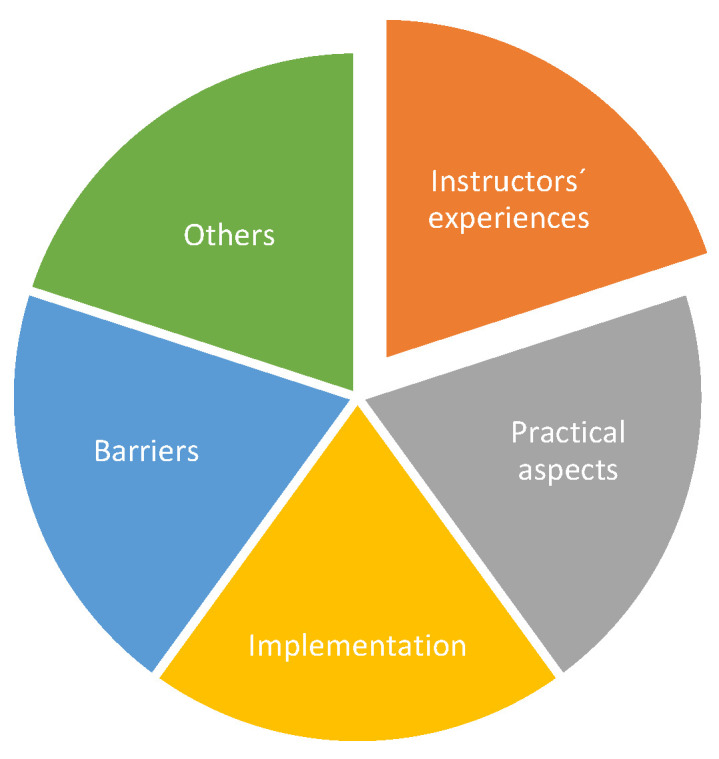
Themes from the focus group interviews of LAC-KT instructors.

The focus group interviews were held in January and April 2023 with 22 LAC-KT instructors. One focus group was held in person with 17 participants and one via Zoom with five participants. The focus groups were moderated by GB and KG. Participating LAC-KT instructors were required to have taught a minimum of two LAC-KTs prior to participating in the focus group interviews. The qualitative results from the interviews of experienced LAC-KT instructors led to the five themes shown in Figure 8 and that will be described in more detail under the different headings.

##### Instructors’ Experiences

The LAC-KT instructors reported many positive experiences with the LAC-KT. These positive experiences included the openness and interest of the children and teenagers in general to talk about death, dying and grief and a willingness to learn about end-of-life care and how to comfort people at the end-of-life. The instructors often reported that the course participants were thankful for the opportunity to talk about these topics in a group.

*Most of them (the participants) say thank you that they were able to say everything they wanted.*

Many instructors reported that participants stated that they had less fear of talking about death and that they felt strengthened to participate in end-of-life care after the LAC-KT. There was also space and time to address a recent death that participants had experienced. Addressing recent losses and a ritual such as lighting a candle or painting a stone seemed to be helpful for the person concerned as well as the whole group.

*We actually had a confirmand who had lost her grandmother one week ago. She came crying to the course and discussed with the priest if she should participate in the course. She was allowed to decide on her own if she wanted to participate. We then lit a candle for her grandmother and she stayed. This led to a great solidarity within the group and she left the course with a smile.*

Negative experiences reported by the LAC-KT instructors were often about problems with parents who did not allow the participation of their children because they thought that they had to protect them. These experiences came from information meetings held before the LAC-KT. Some parents wanted to prevent their child from being confronted with death and dying. Therefore, some parents did not allow the participation of their children in the course. Some instructors also reported problems with time constraints due to the organizational needs of the schools or churches organizing the Confirmation classes. Some would appreciate more time for talking and discussing.

*The lack of time was a problem. In the confirmand teaching program time is very restricted. We were able to get 1.5 h in the first and 1.5 h in the second week. And we won’t get more time. We have to do it (the LAC-KT) within 3 h. This makes it hard for us and sometimes we have to hurry through the course. We would love to have more time.*

##### Practical and Logistical Aspects

The LAC-KT instructors often reported on the practical aspects of planning and delivering the LAC-KT in schools and other venues. The LAC-KT was often held in schools or churches as part of the normal curriculum or Confirmation teaching. In preparation for the delivery of the LAC-KT in schools, many instructors engaged in meetings with the parents of the class in order to inform them about the contents of the LAC-KT and to answer questions of the parents. Some instructors provided written information for the parents about the LAC-KT beforehand. In schools, the instructors usually had to adhere to school rules concerning the number of pupils in a class or the usual school breaks between the lessons. Other practical aspects of providing the LAC-KT were frequently focused on the use of different practical methods in the teaching. Many commented on the effect of using practical exercises and physical activities during the course:

*The children often become less fearful and felt better prepared for participating in end-of-life care, especially after the practical parts of the course. Many of us instructors use an extensive introductory round, often guided by postcards with a variety of images. The practical parts included painting stones, activities around Emoji-cards showing feelings, a jumping sheet to illustrate networking and teamwork and mouth care. Many participants loved the activity with the jumping sheet. This method shows them in practice how networks can support.*

##### Implementation

Most instructors did agree that they usually need a gate-keeper in order to implement the LAC-KT in schools. The gate-keeper may be a religious studies teacher, a school social-worker or a personal contact with school personnel because one of the instructors is a parent of school children in the respective school. The media and newspapers are helpful to raise awareness about the LAC-KT and to interest schools in the Last Aid program.

*You need someone who knows the school. We have often been in the newspaper with our Last Aid Course in the 6th grade. Now the schools contact me. In the beginning I used to write many Emails or letter with information material but there was no response. Sometimes they replied that they would look at it and contact me again.*

##### Barriers

The most negatively influencing barriers for the implementation of the LAC-KT are the teachers and parents who are skeptical about talking about death, dying and grief with children. It seems that many adults want to protect children and teenagers from coming into contact with the topics of death and dying.

*Last time (we did a LAC-KT) the parents of a girl decided that she should not participate. And I thought that this is a bad experience (for the girl). There is so much restraint for talking about this topic (death and dying) that people do not give their children a chance to participate.*

A practical challenge for many of the instructors in schools was big classes with up to 30 pupils. As the LAC-KT usually has a maximum of 20 participants, the question arises of whether the class must be divided into two groups. Another problem is the language barriers for children who do not speak German well enough to understand the teaching and to be able to participate in discussions. These participants can, for example, be refugees or children and teenagers from other countries without adequate understanding and training in the German language. The need for translation by other children can be disturbing and exhausting for both the participants and instructors.

*We have given many courses and within the last half year there are always two or three children who do not speak enough German. That was very exhausting for the children because they did not understand anything.*

##### Others

Other aspects that came up in the focus group interview included the importance of gender-inclusive language in the course materials and the use of the questionnaire as a course evaluation for younger children aged 8–10 years.

## 4. Discussion

This study aimed to explore the views and perspectives of LAC-KT participants and instructors on Last Aid Courses for Kids and Teens. The overall response rate for the LAC-KT participants was 85% as 2543 of the 2996 participants returned the completed questionnaire. 

The main results of the study were: LAC-KTs were very well received by children and teenagers between 7 and 17 years of age. Most of them appreciated the opportunity to talk about death, dying and grief and to learn about end-of-life care and practical Last Aid measures to relieve suffering. The LAC-KT was feasible and very well accepted in different settings as part of the normal school education or Confirmation teaching in churches. Overall, 91% of the participants found the course helpful for everyone and 79% would recommend the course to others. In addition, 2337 of the participants (92%) stated that they learned new things in the course. The findings from this study reinforce that death and grief are ubiquitous topics with little or uncertain knowledge and that the public is lacking awareness and knowledge about palliative care and end-of-life care in general [1,2,3,4,5,6,7]. Thus, the LAC-KT could contribute to improving both awareness and public knowledge about the topics. The results of the current study underline the importance of a training course in Last Aid for Kids and Teens in general and confirm the results of the pilot study on the effects of the LAC-KT in a bigger study population from different regions and settings in Germany [13]. As shown in Figure 5, Figure 6 and Figure 7 and Table 2, the results show that the course format of the LAC-KT is a success, and in many dimensions, similar to the results of the Last Aid Course for adults [12,13]. These findings are also supported by the interviews of LAC-KT instructors who frequently report that the course participants are very thankful for the opportunity to talk about death, dying and grief. In comparison to the course for adults, the LAC-KT differs in the use of more activities and playful tools. The use of activities and playful education methods seems to contribute to the success and great acceptance of the course. In general, the children and teenagers appreciated both the videos and practical parts included in the course, as illustrated by Table 3. Many of the participants liked the practical rituals; e.g., painting stones used in module 4. In this module, stones were painted as an example for a helpful ritual after the death of a loved one. Many of the participants took their painted stone home. This might be one reason for the top ranking of module 4. It is likely that the creative examination of the topic of death and dying has a positive effect on the evaluation, and this is likely to have contributed to the very positive evaluation of modules 3 and 4. It has been stated that engaging in creative activities increases well-being and satisfaction, which in turn has a positive effect on our health. Such activities can also reduce stress and anxiety and increase self-esteem, self-acceptance, self-confidence and self-worth [26]. As death and grief are often associated with fears and uncertainty, creative and playful forms of expression have an anxiety-relieving and liberating effect [27]. The participants rated the playful and creative way of dealing with death and grief very positively. A topic that is otherwise fraught with gravity and fear and tends to be avoided is brought closer to the children in a playful and creative way. The supplementary material includes a video in German language showing the use of activities and play elements in the LAC-KT in a school class during the COVID-19 pandemic [Video S1]. The results from the interviews of the LAC-KT instructors support the importance of the practical activities as a useful approach to discussing and learning about death, dying, grief and palliative care. 

As shown in Table 2, the results of our study suggest that children and teenagers encounter death and dying more often than previously assumed. For example, 84% of the participants had previous experience with death and dying in their family or circle of friends. This implies that it does not make sense not to talk to children and teenagers about death and dying in order to protect them. The LAC-KT instructors frequently reported that the participants were very eager and relieved to be able to talk and learn about death, dying, grief and end-of-life care. This course format therefore represents an important platform for the target group to exchange ideas about death and dying and has the potential to improve public awareness about these topics. An inclusion of the LAC-KT in the school curricula of all schools and the implementation of a nationwide LAC-KT as part of life-long learning approaches could be an important public health palliative care approach with a possible huge impact on end-of-life care in all communities. It has been stated that the Last Aid Course can be seen as the educational basis of compassionate communities [8,12,13] and may help to improve end-of-life care in the community. What is also noteworthy in Table 2 is that the majority of the participants (51%) want to participate in the LAC-KT without the presence of their parents. This may be related to the fact that children grieve and deal with death differently than adults and that many express themselves through art rather than talking [28]. When it comes to death, children see in adults: dismay, crying, apathy, aggression, helplessness, among others. They sense the uncertainty and react to it: every child in its own way. Particularly if the child’s parents themselves are strongly affected by the death, the environment should also pay attention to whether the parents still have the strength to accompany their children. Therefore, participants may prefer to attend the Last Aid Course without their parents in a safe and supportive environment. In order to encourage children, teenagers and adults to talk to each other about death, dying and grief, in some instances, courses for adults have been offered in the same building at the same time but in different rooms with different instructors. In a few cases, a whole family, including teenagers, have participated in an LAC for adults. In this context, it would be interesting to see what concept of a Last Aid Course should be constructed for a family setting. It is frequently stated that men and women often grieve differently. Men are less likely to talk about grief and reach out to others for support. They are more likely to engage in work as a distraction or release. Women are more likely to process grief through talking and reaching out to others [29]. Although there is a danger of using stereotypes connected to gender. this could be one possible reason why the females rated the course better in the “very good” and “good” categories than the males. 

In today’s world, children and young people no longer learn to deal with death, dying and grief. Death, dying and grief are often taboos and are not discussed open in families, workplaces and across the whole of society. As mentioned in the introduction, awareness of palliative care and death literacy are lacking in large parts of the society [1,2,3,4,5,6,7]. The LAC-KT instructors from our study often reported that parents and teachers were skeptical as to whether it was appropriate to talk about death and dying with children and teenagers although many of them already had experience with death. This is important as a barrier for the implementation of Last Aid and other programs that aim to increase death literacy in society. The results of our study can likely contribute to informing parents, teachers and policy makers about the need to address death, dying and palliative care in childhood and adolescence. This may contribute to making it more normal to discuss these issues. The results of the current study show that the children and teenagers appreciated the LAC-KT and that they want to talk about death, dying and grief. Most of them want to learn how to provide practical Last Aid measures and end-of-life care. Many of them are eager to talk and learn about these topics. Several studies have shown that LACs are very well appreciated by the participants and that they can contribute to stimulating the public discussion about death, dying and grief, raise awareness about palliative care in general and empower people to participate in end-of-life care provision in the community and improve death literacy [8,12,13,30,31,32]. Education programs for the public as Last Aid can thereby contribute to compassionate communities by increasing death literacy, developing personal skills and strengthening community action [8,30,31,32].

The results of the current study show that there is a great deal of interest in these topics and that participating children and teenagers can be introduced to these topics in a creative way. The courses enable adolescents to learn more about these topics and to have the opportunity to share opinions and feelings. To the best of our knowledge, this is the first multi-center study showing that a standardized course about death, dying, grief and palliative care is feasible, acceptable and possible to implement in different settings and regions of a country.

Future research is needed about the long-term effects of the course and its effect on increasing the number of supported deaths in the home. It would also be of interest to find out if the children and teenagers show a more empathic behavior towards others in the future after attending an LAC-KT and open discussions about death and dying. 

In summary, the results from the current study can inform the public, health-care personnel, researchers, politicians and decision-makers as both LAC and LAC-KT may have a potential role in reducing the death taboo in societies and to enable caring communities and the improvement of palliative care in the communities. 

### Limitations and Strengths

The most important strength of the study is the high number of participants from different regions of Germany and the high response rate of the LAC-KT participants. The overall response rate was 85% as 2543 of the 2996 participants returned the questionnaire. The results can be seen as representative for the age group as the informants came from different regions and settings of Germany. In schools, the whole class tended to participate in the education despite a few pupils whose parents refused their participation.

An obvious limitation of the study is the timing of the evaluation directly after the course. Therefore, we do not know if the participants are going to use the knowledge and skills acquired in the course in a real life situation. Furthermore, we do not know if their attitude towards life, death and grief and their willingness to provide end-of-life care has changed.

## 5. Conclusions

The LAC-KT is feasible, very well accepted and a welcome opportunity for exchanging and obtaining information about dying, death, grief and palliative care. The findings of the study indicate that LAC-KTs should be offered to all interested children and teenagers and should be included in the school curriculum. Further research on the effects and the implementation of the LAC-KT throughout the school curricula and the long-term effects of the training are needed.

## Figures and Tables

**Table 1:** The contents of the Last Aid Course for Kids and Teens (LAC-KT) [19].

Module Number	Topic	Course Content
Module 1	Dying as a normal part of life	Welcome and introduction First Aid and Last Aid The process of dying What you can do to care
Module 2	Planning ahead	To show feelings are ok! Networks of support Making decisions Advance Directive
Module 3	Relieving suffering	Typical problems and symptoms Caring/-relieving suffering Nutrition at the end of life How to comfort
Module 4	Final goodbyes	Saying goodbye/final fare-well rituals Funeral and various forms of burials Grieving is normal Grief and ways of grieving The right to grieve Questions, Comments

**Table 2:** Participants’ impressions of the Course from the questionnaire (*n* = 2534).

Participants’ Impressions of the Course	Yes	No	No Response
	Number and %	Number and %	Number and %
1. The Last Aid Course is helpful for everyone	2297	164	73
	91%	6%	3%
2. I will recommend the Last Aid Course to others	1994	366	174
	79%	14%	7%
3. All pupils should participate in a Last Aid course	1748	604	182
	69%	24%	7%
4. All parents should participate in a Last Aid course	1666	682	186
	66%	27%	7%
5. Should parents and children visit the course together?	1012	1302	220
	40%	51%	9%
6. I learned something new	2337	139	58
	93%	5%	2%
7. Were the course content easy to understand?	2209	193	132
	87%	8%	5%
8. Have you experienced that anyone in your family/circle of friends has died?	2129	369	36
	84%	15%	1%

**Table 3:** Participants’ Personal Impressions of LAC-KT—The five most frequently mentioned answers (*n* = 2534).

Theme	Number of Participants	Percent
Videos	265	10%
Painting stones	250	10%
Game with swing cloth	244	10%
Mouth care practice	116	5%
Talking	111	4%

## Data Availability

Data are stored on paper questionnaires and are therefore not openly accessible. Only some parts of the data are available on request to the first author due to restrictions of privacy and ethical as well as legal regulations.

## Supplementary Materials

The following supporting information can be downloaded at: https://www.mdpi.com/article/10.3390/children11020224/s1, Table S1: Last Aid Course for kids and teens questionnaire. Video S1: Der letzte Hilfe Kurs für Kids und Teens. Video about the LAC-KT in German language.

## References

[1] McIlfatrick S., Hasson F., McLaughlin D., Johnston G., Roulston A., Rutherford L., Noble H., Kelly S., Craig A., Kernohan W.G. (2013). Public awareness and attitudes toward palliative care in Northern Ireland. BMC Palliat. Care.

[2] McIlfatrick S., Noble H., McCorry N.K., Roulston A., Hasson F., McLaughlin D., Johnston G., Rutherford L., Payne C., Kernohan G. (2014). Exploring public awareness and perceptions of palliative care: A qualitative study. Palliat. Med..

[3] Gopal K.S., Archana P.S. (2016). Awareness, knowledge and attitude about palliative care, in general, population and health care professionals in tertiary care hospital. Int. J. Sci. Study.

[4] Kozlov E., McDarby M., Reid M.C., Carpenter B.D. (2018). Knowledge of Palliative Care Among Community-Dwelling Adults. Am. J. Hosp. Palliat. Care.

[5] Boucher N.A., Bull J.H., Cross S.H., Kirby C., Davis J.K., Taylor D.H. (2018). Patient, Caregiver, and Taxpayer Knowledge of Palliative Care and Views on a Model of Community-Based Palliative Care. J. Pain Symptom Manag..

[6] Westerlund C., Tishelman C., Benkel I., Fürst C.J., Molander U., Rasmussen B.H., Sauter S., Lindqvist O. (2018). Public awareness of palliative care in Sweden. Scand. J. Public Health.

[7] Noonan K. (2018). Death literacy—Developing a tool to measure the social impact of public health initiatives. Ann. Palliat. Med..

[8] Bollig G., Brandt F., Ciurlionis M., Knopf B. (2019). Last Aid Course. An Education For All Citizens and an Ingredient of Compassionate Communities. Healthcare.

[9] Kellehear A. (2013). Compassionate communities: End-of-life care as everyone’s responsibility. QJM Int. J. Med..

[10] Etkind S.N., Bone A.E., Gomes B., Lovell N., Evans C.J., Higginson I.J., Murtagh F.E.M. (2017). How many people will need palliative care in 2040? Past trends, future projections and implications for services. BMC Med..

[11] Sallnow L., Smith R., Ahmedzai S.H., Bhadelia A., Chamberlain C., Cong Y., Doble B., Dullie L., Durie R., Finkelstein E.A. (2022). Report of the Lancet Commission on the Value of Death: Bringing death back into life. Lancet.

[12] Bollig G., Brandt Kristensen F., Wolff D.L. (2021). Citizens appreciate talking about death and learning end-of-life care—A mixed-methods study on views and experiences of 5469 Last aid Course participants. Prog. Palliat. Care.

[13] Bollig G., Hayes Bauer E. (2021). Last Aid Courses as measure for public palliative care education—A narrative review. Ann. Pall. Med..

[14] World Health Organization (1986). The Ottawa Charter for Health Promotion.

[15] Westdeutscher Rundfunk Der Alte Dachs (The Old Badger). https://www.youtube.com/watch?v=YMCg6DRJtrU.

[16] Milford Care Center Bills Story—Talking Together Facing Death and Dying. https://www.youtube.com/watch?v=_5tJGaWjRZk.

[17] Knietzsche und der Tod (Knietzsche and Death). https://www.youtube.com/watch?v=1YGYzBi55JE.

[18] Mark Forster Sowieso (Official Video). https://www.youtube.com/watch?v=jP4-XrbGt3M.

[19] Bollig G., Mainzer K., Fiedler H., Pothmann R. (2020). The last aid course for kids and teens from 8–14 years: -preliminary results from the first pilot test. Hosp. Palliat. Med. Int. J..

[20] Letzte Hilfe Deutschland. https://www.letztehilfe.info.

[21] O’Cathain A., Thomas K., Pope C., Mays N. (2006). Combining qualitative and quantitative methods. Qualitative Research in Health Care.

[22] Sandelowski M. (2000). Whatever happened to qualitative description?. Res. Nurs. Health.

[23] Neergaard M.A., Olesen F., Andersen R.S., Sondergaard J. (2009). Qualitative description–the poor cousin of health research?. BMC Med. Res. Methodol..

[24] Sandelowski M. (2010). What’s in a name? Qualitative description revisited. Res. Nurs. Health.

[25] Malterud K. (2011). Kvalitative Metoder i Medisinsk Forskning.

[26] Fancourt D., Finn S. WHO-Report: What Is the Evidence on the Role of the Arts in Improving Health and Well-Being? A Scoping Review. https://www.artsandhealth.ie/research-evaluation/who-report-what-is-the-evidence-on-the-role-of-the-arts-in-improving-health-and-well-being-a-scoping-review/.

[27] Freeman J., Epston D., Lobovits D. (2000). Ernsten Problemen Spielerisch Begegnen. Narrative Therapie mit Kindern und ihren Familien.

[28] Klinkhammer G. (2012). Trauerbegleitung: Kinder trauern anders. Dtsch. Arztebl..

[29] St. Jude Childrens Research Hospital Gender Differences in the Grieving Process. https://together.stjude.org/en-us/for-families/bereavement/gender-differences-in-grieving.html.

[30] MacAden L., Broadfoot K., Carolan C., Muirhead K., Neylon S., Keen J. (2022). Last Aid Training Online: Participants’ and Facilitators’ Perceptions from a Mixed-Methods Study in Rural Scotland. Healthcare.

[31] Woodworth J. (2023). Building Death Literacy Through Last Aid: An Examination of Agency, Ambivalence and Gendered Informal Caregiving within the Swedish Welfare State. NORA Nord. J. Fem. Gend. Res..

[32] Mills J., Abel J., Kellehear A., Noonan K., Bollig G., Grindod A., Hamzah E., Haberecht J. (2023). The role and contribution of compassionate communities. Lancet.

