# Supplementary material for: “We Want to Talk about Death, Dying and Grief and to Learn about End-of-Life Care”—Lessons Learned from a Multi-Center Mixed-Methods Study on Last Aid Courses for Kids and Teens
Source: Children (Basel). 2024 Feb 9;11(2):224. doi: 10.3390/children11020224 (PMC10887051; doi:10.3390/children11020224)
Supplement: Supplementary file 1 [file children-11-00224-s001.zip › children-2821140-supplementary.pdf]

Table S1: Last Aid Course for kids and teens questionnaire

Evaluation of the course contents
Please set one cross for every course module and one for the course in its whole
(Assessment: 1= very good, 2 = good, 3= satisfactory, 4 = inadequate)

**Theme**	**Rating**			
	**very good**	**good**	**satisfactory**	**inadequate**
1. Dying is a normal part of life				
2. Planning ahead				
3. Relieving suffering				
4. Final goodbyes				
**Assessment oft he course as a whole**				

Please make a cross for yes or no:

1. The Last Aid Course is helpful for everyone ☐ yes ☐ no
2. I will recommend the Last Aid Course to others ☐ yes ☐ no
3. All pupils should participate in a Last Aid course ☐ yes ☐ no
4. All parents should participate in a Last Aid course ☐ yes ☐ no
5. Should parents and children visit the course together? ☐ yes ☐ no
6. I learned something new ☐ yes ☐ no
7. Were the course content easy to understand? ☐ yes ☐ no
8. Have you experienced that anyone in your family/circle of friends has died? ☐ yes ☐ no

Your personal impression (please write in your own words)

1. **What did you like about the course?**

2. **Do you have any open questions?**

Age: **Gender (Male/Female/Divers):** **School grade:**

Please use this space for other comments or suggestions (you may use the back side too)

Thank you very much for your help! **Thank you for your participation!**
